# Optimised Autocalibration Algorithm of Weigh-In-Motion Systems for Direct Mass Enforcement

**DOI:** 10.3390/s20113049

**Published:** 2020-05-27

**Authors:** Piotr Burnos, Janusz Gajda

**Affiliations:** Department of Measurement and Electronics, AGH University of Science and Technology, 30-059 Krakow, Poland; jgajda@agh.edu.pl

**Keywords:** Weigh-In-Motion, WIM, autocalibration, accuracy assessment

## Abstract

Dynamic vehicle weighing systems, also known as Weigh-In-Motion (WIM), are sensitive to factors which interfere with the measurement, including weather and climate conditions. This is a result of the sensitivity of the axle load sensors used in the systems. As a result, a significant change in the precision of weighing can be observed over short periods of time (even less than 1 h). This fact is a deterrent to the use of such systems for direct mass enforcement. In this article, we present a solution for this problem using an optimised autocalibration algorithm. We show the results of simulation studies which we conducted on the proposed algorithm. These were then verified experimentally at an in-road site. We demonstrated that autocalibration of the WIM system allows for effective limitation of the sensitivity of weighing results to interfering factors. This is, however, conditioned on a sufficiently high frequency of reference vehicles crossing the WIM site. The required frequency depends on the speed of change in the concentration of influencing factors.

## 1. Introduction

The need to control the heaviest vehicles and effectively weigh them arises for many reasons. One heavy vehicle with five axles and a gross vehicle weight in excess of the 40 t limit applicable in Poland does more harm through material fatigue to the road surface than 100,000 passenger cars. Heavy traffic of overloaded vehicles is the main cause of premature degradation of roads and bridges, meaning that the useful lifespan of the road surface may be as little as half as what was planned [1,2,3].

In many countries, control efforts are made to eliminate overloaded vehicles from road traffic [4,5,6,7,8]. Such efforts are also made in Poland. Controls are conducted by weighing selected vehicles on scales at static weighing stations or by using low-speed weigh facilities installed at the side of the road. Due to the complexity of the control procedure in Poland and other countries, controlling one vehicle typically takes from 30 to 60 min. The system is thus not effective.

In order to increase the effectiveness of controls, inspectors make use of supplementary results obtained from dynamic Weigh-In-Motion (WIM) systems which play the role of a preselection system [9]. Axle load sensors in such systems are installed directly in the road. The idea of the operation of WIM systems involves measuring the dynamic load which the wheels of the moving vehicle exert on the pavement. Based on this, the static load of each axle of the vehicle is estimated, as is its gross vehicle weight [10].

Currently, more and more research has been carried out on Weigh-In-Motion systems including new construction of axle load sensors [11,12,13,14].

However, due to a variety of interfering factors [15,16,17], the weighing error in WIM systems can amount to from 5% to 10% and, what is worse, this can change while the system is in operation [18]. We have written about this topic previously, demonstrating that the main causes of the instability of the system are changes in pavement temperature and the speed of the weighed vehicle [17]. The low precision of weighing and its variability mean that WIM systems cannot be used for direct mass enforcement.

We are convinced that the currently state of technology does, however, make it possible to construct a WIM system which will meet the requirements set for direct enforcement systems, i.e., where the vehicle weighing error will be maintained over a long period of time at the level of 2%–3%.

The climate conditions in which WIM systems operate are subject to change both on a daily scale and over the course of the seasons [19,20,21]. We discovered that the factors which most impact the precision of weighing in WIM systems are pavement quality and temperature, vehicle speed, wind speed and direction, and the state of the vehicle’s suspension [16,17,22]. The subject of previous research of the authors was primarily the influence of temperature and speed. The load sensors tested were polymer, quartz and bending plate sensors. The tests were carried out in the scope of temperature changes from −10 °C to +30 °C. In the case of polymer sensors, this resulted in a change in weighing result of 50%, for quartz sensors by 7% and for bending plate sensors by 7% [16]. Sensitivity of these sensors to the change of speed of the weighed vehicle is different. For polymer sensors, a speed change in the range from 50 km/h to 90 km/h changes the weighing result by ~10%, for quartz sensors by ~4%–5% and for bending plate sensors by ~2% [17,23]. Studies on the impact of wind direction and power are at an early stage and do not yet allow drawing reliable conclusions. The unevenness of the surface also has a significant impact on weighing accuracy. However, it is limited by formulating requirements that must be met by the road surface at the WIM installation site.

**Figure 1:**
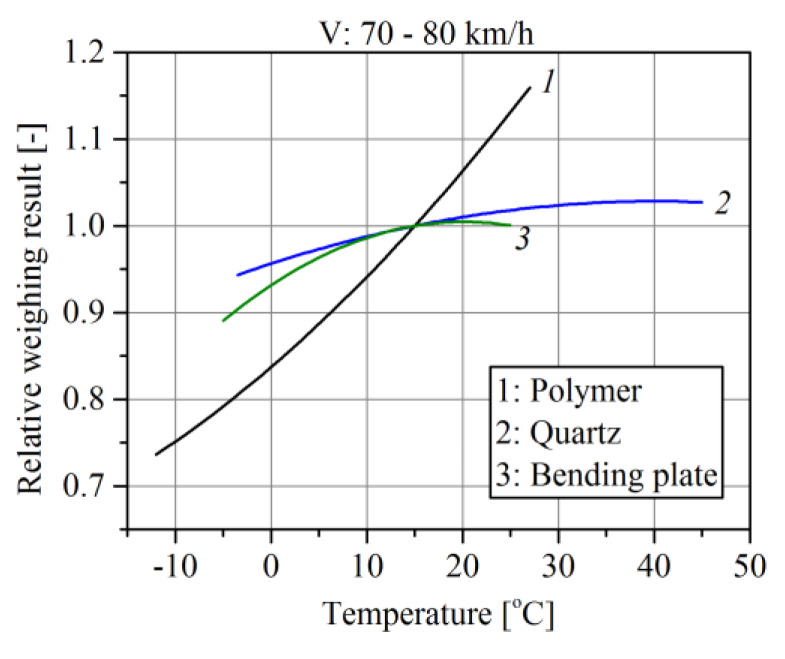
Impact of temperature on vehicle weighing error in Weigh-In-Motion (WIM) systems equipped with sensors manufactured in different technologies.

As can be seen from the characteristics that we developed, which are presented in Figure 1, in the climate conditions which prevail in Poland, in the summer months the daily temperature fluctuation may cause a weighing error of as much as 10%. This phenomenon is observed regardless of the technology used for the manufacture of the axle load sensors.

For obvious reasons, this variability in the precision of weighing is unacceptable for enforcement systems. Unfortunately, such variability in precision cannot be eliminated using traditional, periodic methods of calibration of WIM systems, as these changes take place too quickly.

A solution for this problem was first proposed by Stanczyk [24], who introduced the so-called algorithm of autocalibration of WIM systems. This was further developed and improved by one of the authors of this paper, Burnos, and the results of his research were published in 2012 [25]. Although autocalibration is currently widely used in WIM systems, it does not deliver sufficient weighing precision to satisfy the requirements for enforcement systems. A symptom of malfunctioning of implemented autocalibration algorithms is sensitivity of weighing results to changes in temperature above all. Observing this phenomenon was an inspiration for us to undertake this research. For this reason, in this paper we propose an innovative approach to the construction of an autocalibration algorithm by optimisation of algorithm parameters for the specific environmental conditions which prevail at a given WIM site. The results of our simulation and experimental studies confirm the effectiveness of the solution proposed. The aim of our studies was the quantitative definition of the impact of the parameters of the autocalibration algorithm, the uncertainty of reference value, and of the usage conditions of such a WIM system on vehicle weighing precision which is obtained after optimising algorithm parameters. The results and conclusions presented in this work allow the development of a solution that can significantly reduce the sensitivity of weighing results to slowly changing influencing factors.

The paper is organized as follows. In Section 2, we present the models of the usage conditions that were assumed for the purposes of the simulation studies. In Section 3, we describe the autocalibration algorithm which we propose. Section 4 contains a discussion of the simulation studies which we conducted using the accepted models. In Section 5, we present the results of our experimental studies conducted at an in-road site, which confirm the results formulated previously on the basis of the simulation studies. In the conclusion, we present the basic formulations which result from the studies we conducted.

## 2. Models of Usage Conditions of WIM Systems

We conducted simulation studies of the autocalibration algorithm of WIM systems for established environmental conditions. As the factor which has the greatest impact on changes in precision of weighing in WIM systems is fluctuation of the pavement temperature, it was chosen for the interfering quantity of the measurement. Usage conditions of the WIM system are thus defined by the sensitivity of load sensors to temperature change.

**Figure 2:**
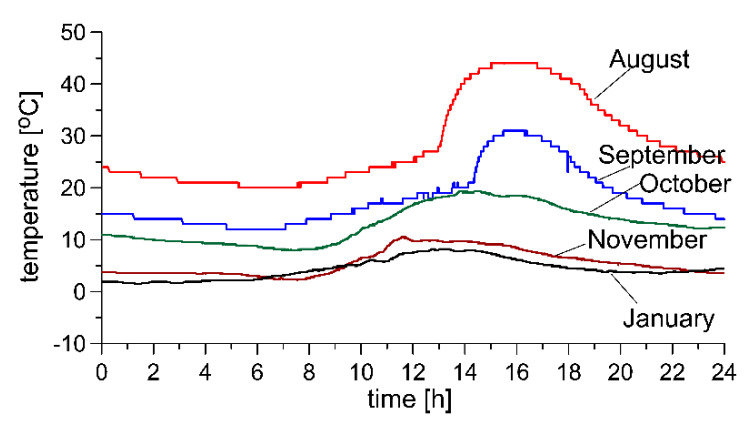
Daily temperature variability of pavement in selected months, in Polish climate conditions.

Temperature variability in the pavement observed in Polish climate conditions is illustrated by the exemplary characteristics shown in Figure 2. As it can be seen, in the summer months the daily change in temperature exceeds 20 °C, or even greater. The range of temperature fluctuation in the scale of the year is substantial and can sometimes exceed 70 °C.

In the model, which we adopted for the purposes of the simulation studies, we assumed that the daily temperature change of the pavement displays a periodic series Ta as shown in Equation (1). Model (1) is simplified compared to the characteristics shown in Figure 2. It allows, however, to easily change the frequency and range of temperature changes in subsequent simulation experiments. It also allows simulation of the WIM system operating conditions much more critical than it results from the characteristics presented in Figure 2. The rest of the paper presents the results of tests analogous to simulation tests, but carried out for experimental data in accordance with Figure 2. The results of these tests confirmed the conclusions formulated based on simulation tests. Arbitrarily set values of these coefficients correspond to the average working conditions of the WIM system, i.e., the period of temperature change equal to 24 h, the range of its changes 0 °C–20 °C, the average value 10 °C. They were changed in subsequent simulation experiments.

(1) $$T_{a}=T_{0}+A_{temp}\sin\left(2\pi tf_{temp}\right),$$

where:

*f_temp_* = 1—frequency of temperature change [cycles/24h],

*A_temp_* = 10—amplitude of temperature change [°C],

*T*_0_ = 10—constant component [°C],

*t*—time [h].

The relation of load sensor sensitivity *C_t_* to temperature depends on the sensor technology (Figure 1). As shown in our previous studies [16] on polymer piezoelectric sensors mounted in asphalt pavements, this relation is described by model (2). Model coefficients (2) were determined as a result of approximation of the experimental temperature characteristic, which describes the dependence of weighing error on temperature. The tests were conducted in the range of temperature (−10 °C–+25 °C). The model coefficients *k_t_ w_t_* and *b_t_* depend on the type and composition of the material from which the road surface is made.

(2) $$C_{t}=b_{t}+k_{t}\cdot 10^{wt\cdot \left(Ta-10\right)}$$

where

*k_t_* = 0.4659, *w_t_* = 0.0098[1⁄°C], *b_t_* = 0.5199,

*T_a_*—current pavement temperature value.

## 3. Autocalibration Algorithm

**Figure 3:**
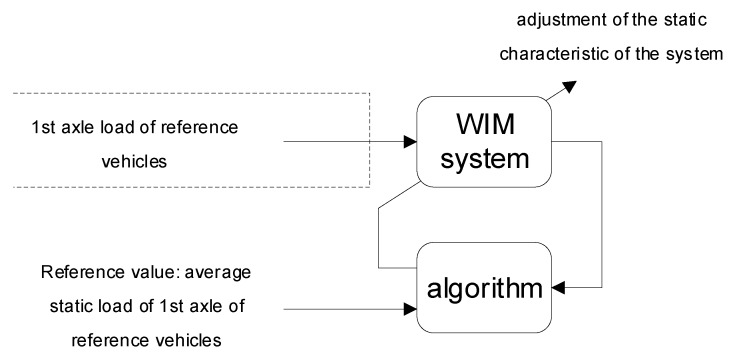
Idea of the autocalibration of WIM system.

The idea of autocalibration of WIM systems involves the adjustment of the static characteristic of the system so as to obtain a correct measurement value for a known reference quantity. For this idea to be implemented, a known reference value must be periodically excited the calibrated system together with simultaneous and automatic detection of this state by the calibrated system. In the case of WIM systems, the role of the reference quantity is played by the axle load of selected classes of vehicles. Reference vehicles are automatically recognised by the system based on features such as their outline and axle configuration. The reference value, in this case the average static axle load of selected reference vehicles, is established based on precise static weighing of a large population of these vehicles. The reference value for autocalibration of the WIM system is the average value of measured loads. The main ideas of this method are presented in Figure 3.

The operation of the autocalibration will be discussed in the context of compensation for the impact of temperature changes on weighing results in a WIM system equipped with polymer load sensors. Changes in temperature cause changes in the sensitivity of the load sensors *C_t_(t)* as shown in the model (2). In a hypothetical WIM system that is not sensitive to changes in temperature, the temperature coefficient should remain constant and equal *C_t_* = 1. The idea of the autocalibration method is to track and adjust the calibration coefficient S of the WIM system to compensate in real time for the impact of changes in *C_t_* on weighing results. In other words, the ideal algorithm should ensure that Equation (3) is met regardless of temperature fluctuations which may occur.

(3) $$C_{t}S=1$$

The source of information on the current temperature coefficient *C_t_(t)* and thus of the value which should be accepted as the calibration coefficient *S* is the result of the measurement of the reference value. For this reason, adjustment of the calibration coefficient *S* can take place only at the moment that a reference vehicle crosses the WIM site. However, the temperature of the pavement fluctuates constantly in the periods between moments of autocalibration of the system. As a result, there are periods of time in between crossings of reference vehicles in which Equation (3) is not met. This is the source of weighing error in WIM systems equipped with an autocalibration algorithm. We refer to this as temperature error. The value of temperature error depends on the temperature characteristics of the pavement/sensor complex, the speed of temperature change, the coefficient of the autocalibration algorithm, and the time gap between successive reference vehicles.

Reference vehicles are selected from classes of vehicles which, due to their construction features, may be automatically recognised by the WIM system. An example of such a class is tractor-trailer vehicles: a 2-axle tractor unit with a 3-axle trailer. The first axle load of vehicles of this class is weakly correlated with the gross vehicle weight, and its relative standard deviation is 0.06. The small random variability of the reference value is a desirable feature in the calibration process (ideally it should be constant). In fact, the reference value changes randomly from vehicle to vehicle.

Due to the random variability of the reference value in subsequent reference vehicles and to random vehicle weighing errors in the WIM system, it is not recommended to radically adjust the calibration coefficient S based on weighing results for only one reference vehicle.

For tracking adjustment of the calibration coefficient *S*, the recursive least squares (RLS) algorithm [3] was used, of which the forgetting factor is optimally derived for minimization of the weighing error (4).

(4) $${\overset{̑}{S}}_{n}={\overset{̑}{S}}_{n-1}+K_{n}\left(\bar{w}-Wd_{n}\cdot {\overset{̑}{S}}_{n-1}\right)$$

(4a) $$b_{n}=1/\left(Wd_{n}\cdot P_{n-1}\cdot Wd_{n}+\lambda \right)$$

(4b) $$K_{n}=P_{n-1}\cdot Wd_{n}\cdot b_{n}$$

(4c) $$P_{n}=\left(P_{n-1}-K_{n}\cdot Wd_{n}\cdot P_{n-1}\right)/\lambda$$

where

$\bar{w}$—the estimation of reference value, calculated based on static weighing of many reference vehicles,

λ—the forgetting factor, with values in the range of (0–1),

$Wd_{n}$—the dynamic weighing result (measurement of the reference value) of the *n*-th reference vehicle for the WIM site,

*n*—the number of the successive reference vehicle which crosses the WIM site,

${\overset{̑}{S}}_{n}$—the estimation of the calibration coefficient of the WIM system indicated in its *n*-th iteration.

The basic features of the algorithm LS with exponential forgetting are well known and described in the literature [3]. In our studies, we focused only on the assessment of the features of the algorithm in the context of its use for autocalibration in WIM systems.

## 4. Simulation Studies of the Autocalibration

The subject of the simulation studies conducted was the quantitative impact of selected parameters of the autocalibration algorithm and of the usage conditions of the WIM system on vehicle weighing error.

The parameters of the autocalibration algorithm which were taken into consideration in the studies included the forgetting factor λ, the reference value $\bar{w}$ (reference value), and random variability of the measurement results σ_1_ (standard deviation of axle load). In the simulation studies conducted, it was assumed that the population of measurement results *Wd_n_* has a normal distribution of the type *N*($\bar{w}$,σ_1_). The parameters which characterise the usage conditions of the system include temperature variability, the sensitivity of the load sensors to temperature, and the frequency with which reference vehicles assed over the sensors *f*_1_. As a measure of the precision of operation of the WIM system, the relative error module ε was assumed.

**Figure 4:**
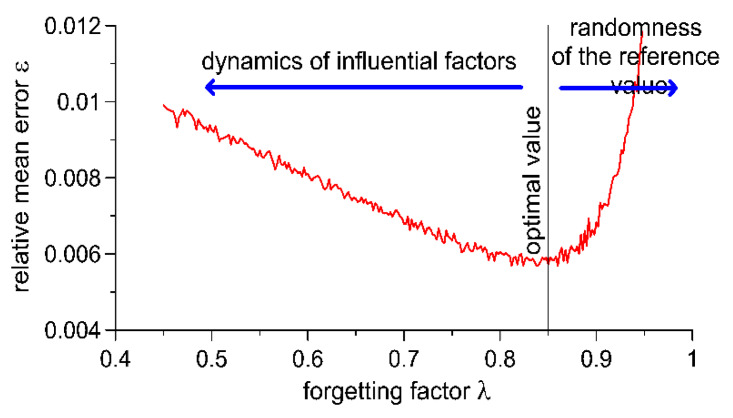
Sample characteristic illustrating the impact of the forgetting factor λ on weighing error.

An increase in the value of the forgetting factor λ generates two effects: First, the autocalibration algorithm becomes less sensitive to random changes in the results of the measurement of the reference value *Wd_n_*. Second, the capability of the algorithm to react to sudden changes in the usage conditions of the WIM system, for example, to sudden changes in temperature, becomes limited. As a result, the characteristic illustrating the impact of the forgetting factor λ on weighing error shows a minimum (Figure 4), whose placement in the coordinate system depends on factors such as the speed of changes in usage conditions (such as temperature), the frequency with which reference vehicles cross the WIM site and random variability of the measurement result of the reference value *Wd_n_*. The result of this is the fact that the value of the forgetting factor λ of the autocalibration algorithm should be selected individually for a given WIM site, taking into consideration the conditions which prevail at the location where the system is installed.

**Figure 5:**
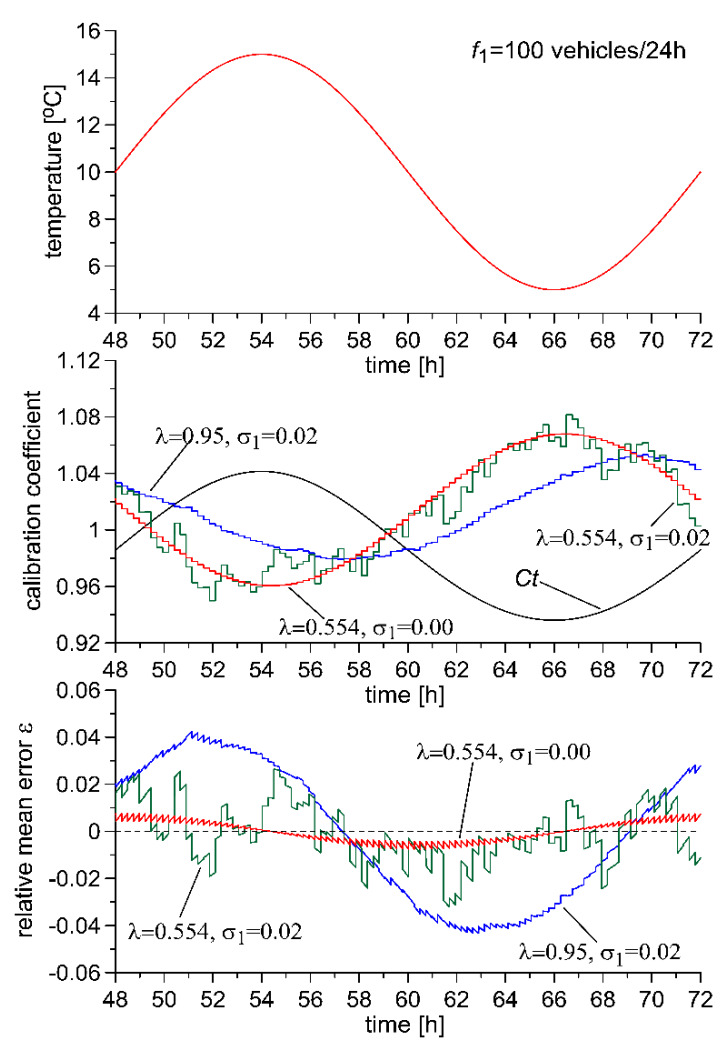
Time variability in vehicle weighing error for two different values of the forgetting factor λ and for a relative standard deviation of the measurement of the reference value of σ_1_ = 0.02 and σ_1_ = 0.0. The frequency of flow of reference vehicles is *f*_1_ = 100 vehicles/24h.

This impact can also be seen in the characteristics presented in Figure 5. It was assumed that during a 24-h period, 100 reference vehicles are weighed at the WIM site, and that the usage conditions of the system are characterised by temperature variability which is represented by Equation (1). Temperature fluctuation causes changes in the sensitivity of the WIM system (*C_t_* coefficient), and without autocalibration of the system, this fluctuation would cause proportional changes in the weighing results. The autocalibration algorithm adjusts the calibration coefficient *S*, which ideally should become a counterphase in relation to *C_t_*. For λ = 0.95, the autocalibration algorithm cannot “keep up with” the fluctuations in temperature, and thus the maximum value of relative weighing error is *ε* = 0.04. This is a value which is not much lower than the value which would appear in the WIM system without autocalibration. Very good results, however, are achieved in dampening random changes in the results of measurement of the reference value. For λ = 0.554, the effects of random variability of the results of measurement of the reference value are clearly visible, while the calibration coefficient does a better job of keeping up with changes in *C_t_* and thus the maximum weighing error is reduced to a value of approximately 0.03. In an ideal case, one in which there is no random variability of the results of measurement of the reference value, the only problem which remains is that of the autocalibration algorithm “keeping up with” the changes in the usage conditions of the system (temperature fluctuation). In such cases, the weighing error decreases monotonically along with the decrease in the value of the forgetting factor. For λ = 0.554, the weighing error reaches a value lower than 0.01.

**Figure 6:**
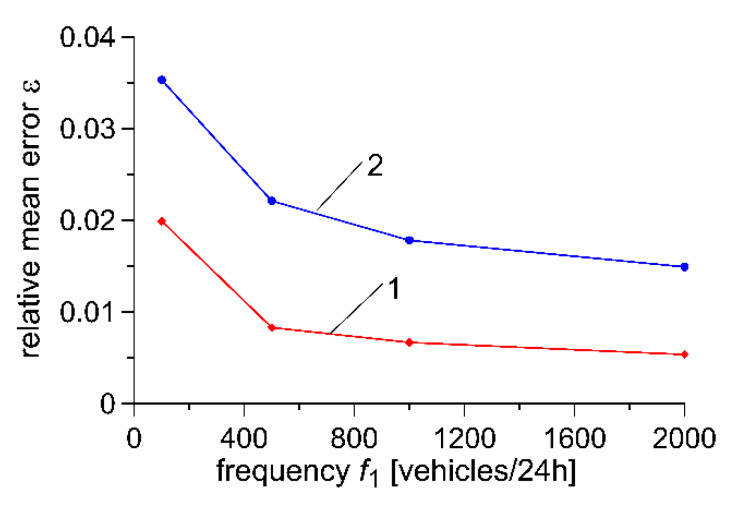
Impact of flow of reference vehicles *f_1_* on vehicle weighing error: 1 – σ_1_ = 0.02, 2 – σ_1_ = 0.10.

Further reduction in the weighing error of vehicles is possible by increasing the frequency of flow of reference vehicles *f*_1_. The impact of frequency *f*_1_ on weighing error is illustrated by the characteristics presented in Figure 6. It must be remembered, however, that this parameter depends on conditions and the structure of traffic flow at the WIM installation site and this may not be subject to optimisation.

### Algorithm Optimisation

**Figure 7:**
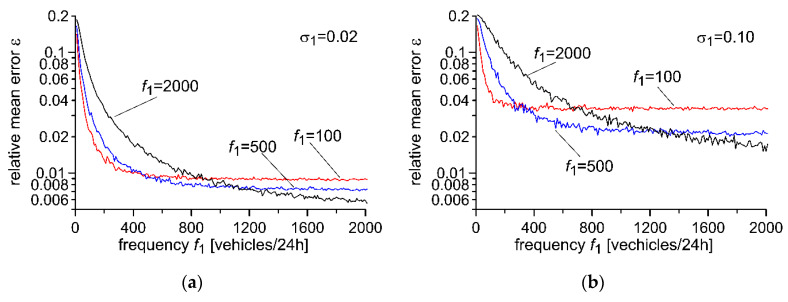
Impact of frequency $f_{1}$ in the area of the optimal point after optimisation of the autocalibration algorithm for a set frequency of flow of reference vehicles $f_{1}$ and for a set relative standard deviation of uncertainty σ_1_ of the reference value. (**a**) Optimisation for σ_1_ = 0.02, (**b**) Optimalization for σ_1_ = 0.10.

**Figure 8:**
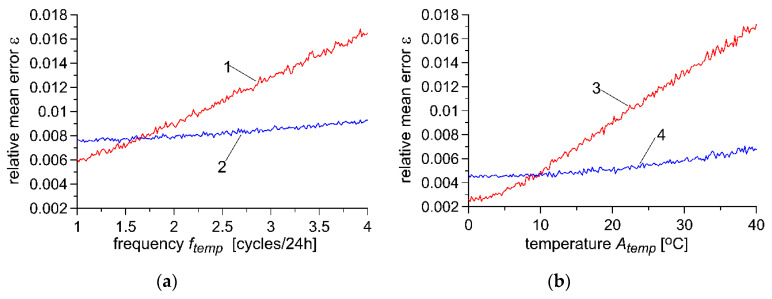
Impact of (**a**) frequency $f_{temp}$ of temperature fluctuation on weighing error for autocalibration algorithm parameters optimised, respectively, for 1 − $f_{temp}=1\left[\mathrm{cycles}/24h\right]$, 2 − $f_{temp}=4\left[\mathrm{cycles}/24h\right]$ as well as the impact of (**b**) the amplitude of $A_{temp}$ temperature change on weighing error for autocalibration algorithm parameters optimised, respectively, for 3 − $A_{temp}\;$ = 5 °C, 4 − $A_{temp}$ =30 °C.

The value of the forgetting factor λ may be selected for known usage conditions of the WIM system using the optimisation method, which aims to minimise the weighing error. The characteristics presented in Figure 7 and Figure 8 illustrate the impact of changes in the parameters of usage of the WIM system in the area of those parameters for which optimisation of the forgetting factor λ has been conducted. Optimisation was conducted for *f*_1_ = 100, 500 and 2000 [vehicles/24h] and for σ_1_ = 0.02 (Figure 7a) as well as for σ_1_ = 0.10 (Figure 7b). In Figure 8, we see impact of frequency $f_{temp}$ of temperature fluctuation on weighing error for optimisation for *f_temp_* = 1 and for 4 [cycles/24h] (Figure 8a), and for *A_temp_ =* 5 °C and 10 °C (Figure 8b).

Results obtained allow us to formulate an important conclusion: optimisation of the autocalibration algorithm for precisely set and stable usage conditions ensures the lowest margin of error. However, when the usage conditions are not known precisely, or when they fluctuate during usage, a lower sensitivity of the WIM system to these changes is ensured by an algorithm optimised for the most critical values. Such a solution is especially useful in WIM systems used for direct mass enforcement, in which weighing uncertainty should not exceed declared values in any conditions. If such values are in fact exceeded, the system should inform the operator of this incident.

## 5. Experimental Studies

Experimental studies of the autocalibration algorithm were conducted based on measurement data obtained from a WIM site equipped with polymer piezoelectric axle load sensors and pavement temperature sensors. Sample characteristics illustrating the daily fluctuation in temperature recorded at the site in selected months of the year are presented in Figure 2. The aim of our study was to verify the results formulated above on the basis of simulation studies.

Just as in the simulation studies, the parameters of the autocalibration algorithm were optimised for recorded temperature fluctuations and for an assumed frequency of flow of reference vehicles and for uncertainty of the measurement of the reference value.

**Figure 9:**
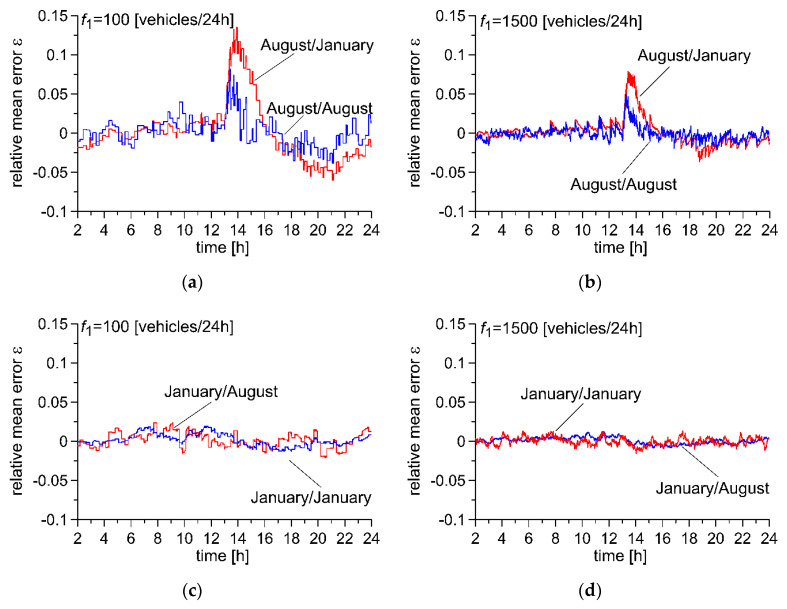
Comparison of precision of WIM systems optimised for various temperature fluctuation ranges. (**a**) *f*_1_ = 100 [vehicles/24h], temperature changes occurring in August, (**b**) *f*_1_ = 1500 [vehicles/24h], temperature changes occurring in August, (**c**) *f*_1_ = 100 [vehicles/24h], temperature changes occurring in January, (**d**) *f*_1_ = 1500 [vehicles/24h], temperature changes occurring in January.

An analysis of the measurement data obtained for a single day for two different frequencies of flow of reference vehicles *f*_1_ was conducted. Data were selected from two months in which substantially different intensities of fluctuation of pavement temperature was observed. The temperature fluctuations observed in the month of August are more rapid and cover a broader range than those observed in January (Figure 2). More critical conditions prevail in the summer months. It thus must be expected that the parameters for the autocalibration algorithm optimised for temperature fluctuations recorded in August will also ensure correct operation of the algorithm in the winter months. A confirmation of this assumption can be obtained by comparing the characteristics presented in Figure 9. The markings “August/August” which appear in this Figure refer to the optimisation of the autocalibration algorithm for temperature fluctuations occurring in the month of August as well as an indication of the trajectory error for temperatures in the month of August. “August/January” refers to the trajectory error for temperature fluctuations occurring in the month of August with optimisation of the autocalibration algorithm, for temperature fluctuations occurring in the month of January.

It can be seen that the use of an autocalibration algorithm ensures the maintenance of similar precision of vehicle weighing, with the exception of a short period of time in which very drastic changes in temperature occurred. It can also be seen that an increase in the frequency of flow of reference vehicles allows this weighing error to be significantly reduced. Moreover, the characteristics presented in Figure 9 confirm the assumptions previously established during simulation studies; the parameters of the autocalibration algorithm optimised for more critical conditions and applied in less critical conditions result only in a slight increase in weighing error (cf. the characteristics “January/August” and “August/January”). The best results are obtained by optimising the parameters of the algorithm for the kind of usage conditions of the WIM system in which it will be subsequently implemented (characteristics “January/January” and “August/August”).

**Figure 10:**
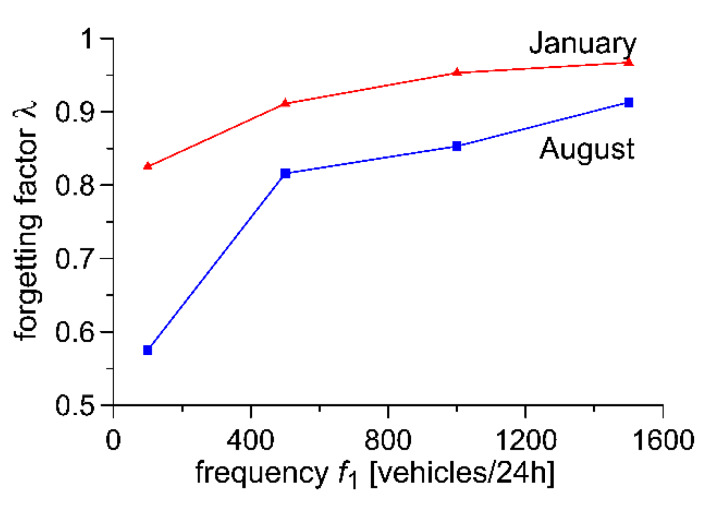
Comparison of precision of WIM systems optimised for various temperature fluctuation ranges.

In terms of the above results, an important factor is the updating of the value of the forgetting factor λ, dependent on the season of the year. In Figure 10, an illustration of the characteristic of the impact of the frequency *f*_1_ of vehicles characteristic for the optimum value of the forgetting factor λ is presented for fluctuations in the temperature of the pavement observed in August and January, respectively. The fluctuations of temperature occurring in August are more critical (rapid changes in temperature over a broad range occurring in a short time). Thus, the optimum values of the forgetting factor are lower than those for temperatures observed in January (when the fluctuations are slower and cover a narrower range). This also ensures the correct operation of the algorithm in other months, during which less critical usage conditions prevail.

## 6. Conclusions

The subject of our study is the precision of vehicle weighing in WIM systems equipped with optimised autocalibration algorithms. We conducted simulation studies of such a system, and subsequently verified the results experimentally. We were able to demonstrate that the autocalibration of a WIM system allows the sensitivity of the weighing results to significantly change in the conditions in which the system is used to be effectively limited. The effectiveness of this limitation of sensitivity is, however, dependent on a sufficiently high frequency of passage of reference vehicles over the WIM site. The required frequency depends on the intensity of changes in influential factors. The algorithm optimisation which we have proposed for specific usage conditions delivered a significant improvement in weighing precision in WIM systems.

The results which we obtained also allow us to formulate the following detailed conclusions.

- The autocalibration algorithm should be optimised for the usage conditions of the WIM system. If these conditions are not stable, then the optimisation should be conducted for the most critical conditions in system usage. In this manner, greater stability of weighing precision can be achieved.
- If the usage conditions of the system are stable, then optimisation conducted for these particular conditions ensures a smaller weighing error. It must be remembered, however, that in such a case the sensitivity of the WIM system to any later changes in usage conditions will be greater.
- WIM systems equipped with an autocalibration algorithm must be fitted with interfering quantity sensors and monitoring of the flow of vehicles crossing the site must be ensured in order to discover any possible changes in the structure of this flow. Changes in the conditions of usage of the system, especially in terms of this factor, can cause significant changes in the precision of weighing which would not be signalled without such monitoring.

Autocalibration algorithms or other methods for compensating for the impact of interfering factors on the measurement (mainly temperature) are universally used in commercial WIM systems. Our research has demonstrated, however, that this algorithm may be substantially improved by optimisation, achieving greater weighing precision than that which currently exists. This is a highly desirable feature for WIM systems for direct enforcement.
